# The Vanderbilt Head and Neck Symptom Survey Brazilian Portuguese version 2.0 (VHNSS 2.0): psychometric properties for patients with head and neck cancer who have undergone radiotherapy

**DOI:** 10.1186/s13104-015-1470-8

**Published:** 2015-10-01

**Authors:** Eliane Marçon Barroso, André Lopes Carvalho, Carlos Eduardo Paiva, Barbara A. Murphy, Bianca Sakamoto Ribeiro Paiva

**Affiliations:** Research Group on Palliative Care and Health-Related Quality of Life (GPQual), Stricto Sensu Graduate Program in Oncology, Barretos Cancer Hospital, Barretos, São Paulo Brazil; Head and Neck Department, Barretos Cancer Hospital, Barretos, São Paulo Brazil; Department of Clinical Oncology, Breast and Gynecology Division, Barretos Cancer Hospital, Barretos, São Paulo Brazil; Department of Medicine, Vanderbilt University Medical Center, Nashville, USA; Fundação Pio XII, Barretos Cancer Hospital, Barretos, São Paulo Brazil; Research and Teaching Institute, 1331, Antenor Duarte Vilella Street, Dr. Paulo Prata, Barretos, São Paulo 14784-400 Brazil

**Keywords:** Head and neck cancer, Psychometric, Oral health, Symptoms, Validation studies

## Abstract

**Background:**

Patients who undergo radiotherapy to treat head and neck cancer can present with several symptoms, including oral ones. The symptoms are usually assessed using instruments to evaluate quality of life. However, these instruments do not really assess oral health outcomes and their functional implications. The VHNSS 2.0 instrument was developed to be used with head and neck cancer patients, and has recently been translated and culturally adapted to be used in Brazil. The purpose of the study was to evaluate the psychometric properties of the VHNSS 2.0 Brazilian Portuguese version.

**Methods:**

Three assessment instruments, the Brazilian Portuguese versions of EORTC QLQ-C30, EORTC H&N 35 and VHNSS 2.0, were answered by 241 head and neck cancer patients, of whom 47 were submitted to the test retest in 5–16 days. The construct validity was assessed through convergent validation (assuming correlations between VHNSS 2.0 and EORTC), and known group analysis (radiotherapy time, site of tumor, staging and surgery). Reliability was evaluated by means of Cronbach’s alpha and test retest using the intraclass correlation coefficient.

**Results:**

241 head and neck cancer patients, median age 58.8, were included in this study. Hypothesized correlations were confirmed, the comparison among the groups showed differences in most of the domains. Reliability for the domains of swallowing solids, dry mouth, mouth pain, mucus, voice, pain and taste/smell presented Cronbach’s alpha values from 0.858 to 0.735 and for the domains of nutrition, swallowing liquids and teeth, 0.618, 0.620 and 0.670 respectively. The test–retest reliability, for the domains of the VHNSS 2.0, measured using intraclass correlation coefficient, ranged from 0.372 to 0.854.

**Conclusion:**

The VHNSS 2.0 Brazilian Portuguese version presented good results for the convergent validation and known-group analyses. It also showed reliability for the Cronbach´s alpha and test retest for most domains.

## Background

Head and neck cancers (HNC) include tumors that affect important anatomical structures, such as the lips, oral cavity, oropharynx, nasopharynx, hypopharynx, larynx, nasal cavity and paranasal sinuses, thyroid gland and salivary glands [1]. Patients undergoing treatment in these regions often have sequelae due to the involvement of vital structures, either due to treatment or disease site. In addition, a considerable number of survivors have adverse effects that may be related to early or delayed treatment [2].

After diagnosis, treatment often results in significant changes, which can be simply subjective for example, pain or functional changes [3] such as breathing, chewing, salivary flow, swallowing and speaking [4]. The assessment of symptoms in patients with HNC can be performed using specific instruments, but often, the symptoms are addressed by quality-of-life (QoL) assessment instruments [5]. It is noteworthy that the instruments available and most commonly used for patients with HNC do not include some important and frequent oral changes. Thus far, some changes have been rarely reported and described, including those related to dental health, mucosal sensitivity and trismus, and their functional implications are not addressed very often either [6].

Considering these aspects, the initial development of the Vanderbilt Head and Neck Symptom Survey version 2.0 (VHNSS 2.0), which is a subjective symptom assessment instrument for HNC patients proposed for use in clinical practice to screen for oral health outcomes, was published in 2012 [6]. It was found to be able to detect the prevalence and severity of oral problems in HNC patients who had undergone radiation [6]. The psychometric proprieties were tested in the same population presenting Cronbach’s alpha ranging from 0.70 to 0.95 [7]. Furthermore, the instrument could detect changes over time in this population [8]. Moreover, in a recent publication, the National Cancer Institute (NCI) recommended that a group of symptoms, including swallowing, oral pain, skin changes, dry mouth, dental health, trismus, taste, excess mucus/saliva, shoulder movement, voice/hoarseness and some QoL domains (social and functional), should be evaluated in clinical trials because they are relevant for most HNC patients [9].

VHNSS 2.0 was developed in English and has recently been translated and culturally adapted into Brazilian Portuguese [10]. This study aimed to evaluate the psychometric properties of VHNSS 2.0 Brazilian Portuguese version.

## Methods

### Design and study site

This was a descriptive, cross-sectional study with an assessment instrument validation methodology, using the STROBE guidelines for reporting observational studies [11], carried out at the Department of Head and Neck Cancer, Barretos Cancer Hospital, São Paulo, Brazil. Patients were included from September 2013 to August 2014.

### Study population

The population comprised patients older than 18 with a history of HNC (oral cavity, hypopharynx, oropharynx and larynx), whose radiotherapy treatment ended 6 months or more prior to the study, and who could read. Important cognitive changes that would prevent participants from responding to the assessment instruments were considered as exclusion criteria. These cognitive changes were identified by means of medical records and the perception of the researcher.

### Ethical statement

This study was approved by the Research Ethics Committee of the Barretos Cancer Hospital (644/2012) and was developed according to the ethical principles of the Declaration of Helsinki and National Health Council (Brazil), Resolution 466/2012. All subjects participated voluntarily and signed an informed consent form.

### Data collection

This step was performed by a single properly trained researcher (EMB) who identified eligible patients and invited them to participate. Socio-demographic characteristics, such as gender, race, civil status and education were self-reported and clinical characteristics (histological type, staging, tumor site, and treatment/s) were collected by means of medical records. The instruments were applied individually in a reserved environment, and each patient was given a choice between self-administering the instrument and having the instrument applied by the researcher. When applied by the interviewer, the questions and answers were read and care was taken not to provide any explanations. If the patients did not respond for any reason, the item was left blank and recorded as a non-response. The time required for completing the instrument was measured using a stopwatch.

### Data collection instruments

Three instruments were used: the Brazilian Portuguese versions of the instruments Vanderbilt Head and Neck Symptom Survey version 2.0 [6], EORTC QLQ C30 [12] and EORTC H&N 35 [13].

#### Vanderbilt Head and Neck Symptom Survey version 2.0 (VHNSS 2.0)

It is an instrument developed specifically for patients with HNC and is intended to evaluate symptoms and oral changes in patients who have undergone radiotherapy. It was developed in 2012 [6] and comprises 10 domains and three single items: nutrition (four items), swallowing solids (eight items), swallowing liquids (two items), dry mouth (five items), mouth pain (six items), general pain (three items), mucus (four items), voice/communication (three items), hearing (one item), taste/smell (six items), teeth (four items), neck range of motion (one item) and trismus (one item). Response choices range from 0 (none) to 10 (severe) so that, the higher the score is, the greater the intensity of symptoms will be. The mean of each domain is calculated only if there is a response for at least half of the items. Items receiving a “not applicable” response are treated as a non-response in the score calculation. The time reference is in relation to the previous week.

#### EORTC QLQ C 30

It is a questionnaire evaluating cancer-specific QoL that has been previously validated for use in Brazil [14, 15] and comprises 30 items, including five functional scales, three symptom scales, an overall health scale and some individual items related to symptoms commonly reported by cancer patients, with responses graded on a Likert scale varying from 0 to 4 points. For QoL-related and overall health status items, the responses are graded on a 7-point Likert scale. Scores range from 0 to 100, where 0 represents the worst health status and 100 represents the best health status; this is in contrast to the way in which the symptom scales work, where higher scores represent a higher level of symptoms and a worse QoL [12]. Cronbach’s alpha in this study was calculated to be 0.895.

#### EORTC H&N 35

The EORTC H&N 35 [13] was developed by the European Organization for Research and Treatment of Cancer (EORTC). It is specific for patients with HNC and is complementary to QLQ C30. It contains 35 items that evaluate seven domains: pain, swallowing, senses (taste and smell), speech, social eating, social contact, and sexuality, as well as single specific items related to dental problems, trismus, dry mouth, sticky saliva, coughing, feeling ill, use of pain killers, use of nutritional supplements, use of feeding tube and weight gain/loss. Thirty of the responses were graded on a Likert scale ranging from 1 to 4, and five questions required a yes or no response. High scores represent high symptomatology. The questions are related to events in the previous week. Cronbach’s alpha in this study was calculated to be 0.885.

### Validation process stages

#### Psychometric properties

Internal consistency was measured using Cronbach’s α, considering a value between 0.70 and 0.95 as acceptable [16].

The reproducibility of the VHNSS 2.0 instrument was evaluated by the ICC, considering values ≥0.7 as acceptable [16]. This was performed over a period of 1–2 weeks, which could vary up to 2 days, in the same setting as the baseline measures. Once the subjects were follow up patients and did not have to come back to the hospital very often, the ones included were the ones who came, for any reason, within this time interval. As the retest should be performed on clinically stable patients, the performance status (ECOG) was evaluated at both time points to confirm clinical stability.

Construct validity was assessed using hypothesis testing. For convergent validity, correlations were assumed to exist between scores for nutrition, swallowing solids, swallowing liquids, dry mouth, mouth pain, voice, general pain, taste/smell and trismus measured by VHNSS 2.0, and scores for social eating, swallowing, dry mouth, pain, speech problems, pain, sense problems and opening mouth as measured by EORTC H&N 35. Correlations between the general pain and mouth pain domains of VHNSS 2.0 and pain domain of EORTC QLQ C-30 were also assumed. Correlations >0.4 were considered as acceptable [17].

In the known-groups analysis, the groups were compared using the mean (standard deviation) symptoms of each domain as measured by VHNSS 2.0, to assess whether the instrument could discriminate between the patient groups. It was assumed that those who completed radiotherapy between 6 and 12 months versus >12 months; those who underwent surgical treatment versus no surgical treatment, and those diagnosed as stage I/II versus III/IV would all differ regarding the scores of the instrument. An additional exploratory analysis was performed to assess whether the instrument could discriminate among groups of patients with diseases at different sites, comparing those with diseases in the oral cavity/oropharynx to those with disease in the hypopharynx/larynx.

These differences were assumed, since the scores of symptom items measured using VHNSS 2.0 tend to improve over time, considering early, mid and late recovery, post chemo-radiation [18]. Furthermore, patients with advanced stages were expected to present higher symptom scores [13, 19]. It was also expected that patients who had undergone surgery would present problems related to the procedure, such as mouth opening [20]. Moreover, the differences among sites were assumed because oral cancer patients may present problems with teeth, trismus and pain; pharynx cancer patients usually present alterations related to swallowing, social eating and stick saliva, whereas larynx cancer patients report higher scores in the voice and cough scales [13].

Missing information was evaluated considering the number of non-responses per item, with values of up to 4 % being considered acceptable [17].

### Statistical analyses

All the data were analyzed using the IBM SPSS Statistic 21 statistical program and Software R program, adopting a 5 % significance level. Cronbach’s alpha coefficient was used to calculate internal consistency, and the ICC was used for test–retest evaluation. Convergent validity correlations were evaluated using Pearson’s correlation coefficient. For the known-groups analysis, the groups were compared using the nonparametric Mann–Whitney U-test.

The sample size was calculated using the Cronbach’s alpha coefficient expected by the researcher (α = 0.7), under the null hypothesis (α = 0.6), considering a 5 % significance level, and 85 % test power [21, 22], resulting in a sample of 224 patients. For the retest, the intraclass correlation coefficient (ICC) expected by the researcher (ρ = 0.85) was used, under the null hypothesis (ρ = 0.7), with a 5 % significance level, and 85 % test power, resulting in 47 patients.

## Results

Two hundred sixty-five patients were invited to participate in the study, of whom 19 refused, and five could not respond to the instrument because they presented with important cognitive changes, leaving 241 participants. The median age of the participants was 58.8 (range 33.49–88.55) years. All the patients preferred the interview to be applied by the researcher, and the median time of application of VHNSS 2.0 and EORTC H&N 35 was 8 (range 4–17) and 6 (range 2–17) minutes, respectively. The median time between the end of radiotherapy and interview was 2 (range 0–23) years. The socio-demographic and clinical characteristics are described in Table 1.

**Table 1:** Description of sociodemographic and clinical characteristics

Characteristics	N (%)
Gender	
Female	32 (13.3)
Male	209 (86.7)
Race	
White	144 (59.8)
Black	16 (6.6)
Other races	81 (33.6)
Civil status	
Single	32 (13.3)
Married	164 (68.0)
Separated/divorced	32 (13.3)
Widowed	13 (5.4)
Education	
<8 years	161 (67.1)
≥8 to <11 years	26 (10.8)
≥ 12 years	53 (22.1)
Family monthly income^a^	
<1	11 (4.7)
≥1 and <3	173 (73.3)
≥3 and <6	40 (16.9)
≥6	12 (5.1)
Professional status^b^	
Inactive	183 (75.9)
Active	58 (24.1)
Teeth	
No	92 (38.2)
Yes	149 (61.8)
Histological type	
SCC	234 (97.1)
Other	7 (2.9)
TNM	
0	1 (0.4)
I	37 (15.9)
II	32 (13.7)
III	77 (33)
IV	86 (36.9)
Tumor site	
Oral cavity	57 (23.7)
Hypopharynx	21 (8.7)
Oropharynx	61 (25.3)
Larynx	102 (42.3)
ECOG	
0	172 (71.4)
1	67 (27.8)
2	2 (0.8)
Type of treatment	
Radiation	40 (16.39)
Radiation + chemotherapy	85 (35.26)
Radiation + chemotherapy + surgery	51 (21.16)
Radiation + surgery	65 (26.97)
Full denture	
No	107 (44.4)
Yes	134 (55.6)

*SSC* squamous cell carcinoma, *TNM* classification of malignant tumors, *ECOG* Eastern Cooperative Oncology Group

^a^Brazilin minimum wages (R$)

^b^Professionally active: had a job, inactive: unemployed, retired, or on sick leave

### Descriptive analysis of the VHNSS 2.0 items

Table 2 shows the frequency and severity of symptoms measured by the VHNSS 2.0 instrument, graded as no symptoms (0), mild (1–4), moderate (5–6) and severe (>7) [23]. Items from the swallowing solids, dry mouth, mucus, taste/smell, voice and teeth domains had higher percentages of moderate to severe scores. The percentage of missing items was 0.21 % (25/11568).

**Table 2:** Frequency and severity of scores measured by VHNSS 2.0 and Cronbach’s alpha

Domains/items descriptions	*N (Missing)*	VHNSS 0	VHNSS 1–4 mild	VHNSS 5–6 moderate	VHNSS >7 severe	Cronbach’s alpha	Alpha if item deleted
		n (%)	n (%)	n (%)	n (%)	(95 % CI)	
Nutrition						0.618 (0.532–0.691)	
Weight loss	239 (2)	201 (84.1)	25 (10.5)	7 (2.9)	6 (2.5)		0.571
Appetite loss	241 (0)	190 (78.8)	15 (6.2)	20 (8.2)	16 (6.6)		0.542
Supplement use	240 (1)	187 (77.9)	5 (2.1)	12 (5.0)	36 (15.0)		0.656
Trouble maintaining weight	241 (0)	188 (78.0)	17 (7.1)	20 (8.3)	16 (6.6)		0.418
Swallowing solids						0.858 (0.829–0.883)	
Trouble eating solids	241 (0)	59 (24.5)	47 (19.5)	69 (28.6)	66 (27.4)		0.842
Food gets stuck in mouth	241 (0)	125 (51.9)	40 (16.6)	50 (20.7)	26 (10.8)		0.831
Food gets stuck in throat	241 (0)	129 (53.5)	53 (22.0)	34 (14.1)	25 (10.4)		0.837
Chokes on solids	241 (0)	158 (65.6)	44 (18.3)	27 (11.2)	12 (5.0)		0.842
Cough after swallow	241 (0)	170 (70.5)	38 (15.8)	23 (9.5)	10 (4.1)		0.856
Swallowing takes effort	241 (0)	146 (60.6)	41 (17.0)	32 (13.3)	22 (9.1)		0.829
Eating takes longer	240 (1)	106 (44.2)	40 (16.7)	57 (23.8)	37 (15.4)		0.823
Sensitivity to acidic, spicy or hot foods	241 (0)	118 (49.0)	36 (14.9)	37 (15.4)	50 (20.7)		0.862
Swallowing liquids						0.620 (0.511–0.705)	
Trouble drinking liquids	241 (0)	204 (84.6)	20 (8.3)	10 (4.1)	7 (2.9)		_
Chokes on liquids	241 (0)	185 (76.8)	36 (14.9)	12 (5.0)	8 (3.3)		_
Dry mouth						0.840 (0.806–0.870)	
Dry mouth	241 (0)	47 (19.5)	45 (18.7)	56 (23.2)	93 (38.6)		0.803
Difficulty chewing	240 (1)	92 (38.3)	43 (17.9)	46 (19.2)	59 (24.6)		0.779
Difficulty sleeping	241 (0)	171 (71.0)	29 (12.0)	17 (7.1)	24 (10.0)		0.853
Difficulty speaking	240 (1)	122 (50.8)	38 (15.8)	39 (16.3)	41 (17.1)		0.804
Sensitivity to dryness	241 (0)	123 (51.0)	39 (16.2)	44 (18.3)	35 (14.5)		0.791
Mouth pain						0.829 (0.783–0.868)	
Sores cause pain	241 (0)	214 (88.8)	12 (5.0)	8 (3.3)	7 (2.9)		0.801
Trouble swallowing	241 (0)	208 (86.3)	9 (3.7)	15 (6.2)	9 (3.7)		0.778
Trouble speaking	240 (1)	208 (86.7)	12 (5.0)	12 (5.0)	8 (3.3)		0.780
Sensitivity of mouth/throat	241 (0)	197 (81.7)	19 (7.9)	14 (5.8)	11 (4.6)		0.788
Altered food choices	241 (0)	201 (84.6)	9 (3.7)	14 (5.8)	14 (5.8)		0.811
Difficulty brushing teeth	153 (0)	141 (92.2)	2 (1.3)	5 (3.3)	5 (3.3)		0.842
Mucus						0.743 (0.685–0.792)	
Mucus/phlegm	240 (1)	116 (48.3)	51 (21.3)	45 (18.8)	28 (11.7)		0.716
Choking	241 (0)	204 (84.6)	17 (7.1)	8 (3.3)	12 (5.0)		0.685
Difficulty swallowing	241 (0)	203 (84.2)	16 (6.6)	13 (5.4)	9 (3.7)		0.689
Sleep affected	240 (1)	198 (82.5)	20 (8.3)	15 (6.3)	7 (2.9)		0.653
Voice/communication						0.735 (0.671–0.789)	
Trouble speaking	240 (1)	135 (56.3)	37 (15.4)	49 (20.4)	19 (7.9)		0.646
Hoarse voice	239 (2)	97 (40.6)	59 (24.7)	48 (20.1)	35 (14.6)		0.742
Trouble being understood	238 (3)	137 (57.6)	33 (13.9)	37 (15.5)	31 (13.0)		0.544
Taste/smell						0.823 (0.786–0.856)	
Taste altered	239 (2)	140 (58.6)	31 (13.0)	41 (17.2)	27 (11.3)		0.756
Decreased desire to eat	241 (0)	177 (73.4)	21 (8.7)	26 (10.8)	17 (7.1)		0.757
Altered food choices	240 (1)	181 (75.4)	20 (8.3)	23 (9.6)	16 (6.7)		0.788
Decreased food eaten	241 (1)	167 (69.3)	28 (11.6)	31 (12.9)	15 (6.2)		0.766
Sense of smell changed	240 (0)	177 (73.8)	23 (9.6)	22 (9.2)	18 (7.5)		0.853
Altered food choices	241 (0)	219 (90.9)	9 (3.7)	9 (3.7)	4 (1.7)		0.823
Teeth						0.670 (0.573–0.749)	
Difficulty chewing	226 (1)	79 (35.0)	36 (15.9)	55 (24.3)	56 (24.8)		0.592
Teeth sensitive to hot, cold, sweet foods	147 (1)	75 (51.0)	23 (15.6)	23 (15.6)	26 (17.7)		0.545
Teeth feel looser	147 (1)	108 (73.5)	15 (10.2)	10 (6.8)	14 (9.5)		0.566
Cracking/chipping teeth	147 (1)	105 (71.4)	16 (10.9)	18 (12.2)	8 (5.4)		0.683
General pain						0.820 (0.776–0.856)	
Average pain level	241 (0)	173 (71.8)	41 (17.0)	17 (7.1)	10 (4.1)		0.610
Worst pain level	240 (1)	174 (72.5)	30 (12.5)	16 (6.7)	20 (8.3)		0.639
Pain causing difficulty sleeping	240 (1)	212 (88.3)	9 (3.8)	8 (3.3)	11 (4.6)		0.927
Trismus							
Limited mouth opening	241 (0)	173 (71.8)	26 (10.8)	26 (10.8)	16 (6.6)		–
Neck							
Limitations in neck/shoulder movement	241 (0)	173 (71.8)	30 (12.4)	24 (10.0)	14 (5.8)		–
Hearing							
Hearing problems	241 (0)	166 (68.9)	24 (10.0)	23 (9.5)	28 (11.6)		–

In Table 3, it can be observed that the most affected domain was dry mouth, with a mean (standard deviation [SD]) score of 3.38 (2.72), followed by swallowing solids (mean = 2.63, SD = 2.22), voice (mean = 2.59, SD = 2.60) and teeth (mean = 2.37, SD = 2.28. The least affected domains were mouth pain, swallowing liquids and pain, with mean (SD) scores of 0.76 (1.52), 0.86 (1.74) and 1.13 (2.10), respectively.

**Table 3:** Descriptive analyses of VHNSS 2.0 domains

VHNSS 2.0 domains	Mean	Standard deviation	Minimum	Median	Maximum
Nutrition	1.24	1.89	0.00	0.00	9.25
Swallowing solids	2.63	2.22	0.00	2.25	9.88
Swallowing liquids	0.86	1.74	0.00	0.00	9.00
Dry mouth	3.38	2.72	0.00	2.80	10.0
Mouth pain	0.76	1.52	0.00	0.00	7.67
Mucus	1.30	1.87	0.00	0.50	10.00
Voice	2.59	2.60	0.00	1.67	10.00
General pain	1.13	2.10	0.00	0.00	10.00
Taste/Smell	1.46	1.97	0.00	0.50	9.17
Teeth	2.37	2.28	0.00	2.00	10.00
Trismus	1.54	2.72	0.00	0.00	10.00
Neck	1.44	2.59	0.00	0.00	10.00
Hearing	1.78	3.05	0.00	0.00	10.00

### Construct validity

#### Convergent validity

As expected, the hypothetical correlations between the VHNSS 2.0 and EORTC QLQ C30, and VHNSS 2.0 and EORTC H&N 35 domains were confirmed, presenting correlations >0.4 (Table 4).

**Table 4:** Correlation coefficient between VHNSS 2.0 and the EORTC QLQ C30 and EORTC H&N 35 (convergent validity)

VHNSS 2.0 domains	Instruments	Domains/items	Correlation coefficient (r)	95 % CI
Nutrition	EORTC QLQ C30	Appetite loss	0.601*	(0.514–0.676)
	EORTC H&N 35	Social eating^a^	0.537*	(0.440–0.622)
Swallowing solids	EORTC H&N 35	Nutritional supplements	0.667*	(0.591–0.732)
	EORTC H&N 35	Swallowing^a^	0.756*	(0.696–0.805)
	EORTC H&N 35	Social eating	0.648*	(0.568–0.716)
Swallowing liquids	EORTC H&N 35	Swallowing^a^	0.470*	(0.365–0.563)
Dry mouth	EORTC H&N 35	Swallowing^a^	0.597*	(0.509–0.673)
	EORTC H&N 35	Dry mouth^a^	0.713*	(0.645–0.770)
	EORTC H&N 35	Sticky saliva	0.543*	(0.447–0.627)
Mouth pain	EORTC QLQ C30	Pain^a^	0.485*	(0.382–0.576)
	EORTC H&N 35	Pain^a^	0.659*	(0.581–0.725)
	EORTC H&N 35	Swallowing	0.578*	(0.488–0.657)
	EORTC H&N 35	Social eating^a^	0.418*	(0.308–0.518)
Mucus	EORTC H&N 35	Cough	0.467*	(0.362–0.560)
Voice	EORTC H&N 35	Speech problems^a^	0.739*	(0.676–0.792)
General pain	EORTC QLQ C30	Pain^a^	0.583*	(0.492–0.660)
	EORTC H&N 35	Pain^a^	0.572*	(0.480–0.651)
	EORTC H&N 35	Pain killers	0.545*	(0.450–0.628)
Taste/smell	EORTC H&N 35	Senses problems^a^	0.667*	(0.590–0.731)
Teeth	EORTC H&N 35	Teeth	0.570*	(0.449–0.670)
Trismus	EORTC H&N 35	Opening mouth^a^	0.748*	(0.687–0.799)

*VHNSS 2.0* Vanderbilt Head and Neck Cancer Symptom Survey version 2.0, : European Organization for Research and Treatment of Cancer, *r* Pearson’s correlation coefficient, *95* *% CI* 95 % confidence interval

* p < 0.001

^a^Correlations assumed a priori; the remaining correlations were additional findings

#### Known-groups validity

The Known-groups analysis, considering time of therapy completion, stage of disease, tumor site and surgical or non-surgical treatment, showed that the instrument could discriminate between patient groups, as shown in Table 5.

**Table 5:** Mean comparison of symptoms measured by VHNSS 2.0 between patients groups (known-groups analysis)

Domains	Radiotherapy			Staging			Surgery			Tumor site		
	> 6 and ≤12 months	>12 months	p value	I/II	III/IV	p value	No	Yes	p value	Oral cavity/oropharynx	Hypopharynx/larynx	p value
	Média (DP) (n = 52)	Média (DP) (n = 185)		Média (DP) (n = 70)	Média (DP) (n = 163)		Média (DP) (n = 125)	Média (DP) (n = 116)		Média (DP) (n = 118)	Média (DP) (n = 123)	
Nutrition	2.11 (2.26)	1.02 (1.72)	*<0.001*	0.96 (1.99)	1.34 (1.85)	*0.005*	1.18 (2.00)	1.30 (1.74)	0.295	1.88 (2.16)	0.63 (1.33)	*<0.001*
Swallowing solids	3.40 (2.19)	2.43 (2.19)	*0.002*	1.88 (2.41)	2.92 (2.9)	*<0.001*	2.32 (2.33)	2.96 (2.04)	*0.002*	3.32 (2.29)	1.95 (1.92)	*<0.001*
Swallowing liquids	0.95 (1.68)	0.84 (1.77)	0.354	1.04 (1.83)	0.78 (1.69)	0.052	0.80 (1.79)	0.93 (1.69)	0.318	1.07 (1.94)	0.65 (1.49)	0.103
Dry mouth	5.00 (2.52)	2.95 (2.60)	*<0.001*	2.31 (2.64)	3.78 (2.66)	*<0.001*	3.22 (2.78)	3.55 (2.65)	0.238	4.31 (2.81)	2.47 (2.29)	*<0.001*
Mouth pain	1.23 (1.84)	0.63 (1.40)	*0.002*	0.72 (1.61)	0.77 (1.46)	0.449	0.76 (1.70)	0.76 (1.30)	0.116	1.12 (1.76)	0.41 (1.13)	*<0.001*
Mucus	1.58 (1.77)	1.23 (1.91)	0.111	1.12 (1.95)	1.37 (1.83)	0.227	1.26 (2.07)	1.33 (1.62)	0.076	1.37 (1.96)	1.21 (1.76)	0.893
Voice	3.57 (2.61)	2.33 (2.55)	*0.001*	2.17 (2.33)	2.81 (2.71)	0.200	2.47 (2.85)	2.72 (2.30)	0.157	2.54 (2.65)	2.62 (2.55)	0.462
General pain	1.70 (2.39)	1.00 (2.01)	*0.016*	0.87 (2.02)	1.26 (2.16)	0.055	1.27 (2.29)	0.98 (1.87)	0.644	1.36 (2.24)	0.90 (1.93)	0.051
Hearing	2.08 (3.12)	1.69 (3.03)	0.125	1.66 (2.98)	1.71 (3.01)	0.893	1.97 (3.23)	1.57 (2.84)	0.33	1.76 (3.02)	1.78 (3.08)	0.983
Taste/smell	2.51 (2.18)	1.18 (1.82)	*<0.001*	0.95 (1.72)	1.68 (2.04)	*0.001*	1.40 (2.07)	1.53 (1.85)	0.129	1.88 (2.10)	1.05 (1.74)	*<0.001*
Trismus	2.02 (3.03)	1.41 (2.64)	0.181	1.34 (2.93)	1.61 (2.64)	0.167	1.23 (2.66)	1.86 (2.76)	*0.018*	2.62 (3.17)	0.48 (1.62)	*<0.001*
Neck	1.44 (2.58)	1.45 (2.62)	0.900	0.97 (2.14)	1.56 (2.65)	0.118	1.17 (2.36)	1.72 (2.80)	0.105	1.82 (2.95)	1.06 (2.13)	0.055
Teeth*	2.36 (2.50)	2.31 (2.12)	0.812	1.90 (1.95)	2.67 (2.44)	0.087	1.88 (1.95)	2.99 (2.51)	*0.005*	2.94 (2.50)	1.97 (2.02)	*0.018*

Mann–Whitney Test. * Teeth domain: sample size varies depending on the subgroups (Staging I/II = 51, III/IV = 92; Oral cavity/oropharynx = 60, Hypopharynx/Larynx = 87, Radiotherapy >6 and ≤12 months = 31, >12 months = 114; Non-surgical = 82 and surgical = 65)

Statistically significant p values at the 0.05 level are in italics

### Reliability

#### Internal consistency

Most domains had Cronbach’s α values ≥0.70, except for nutrition (α = 0.618), swallowing liquids (α = 0.620) and teeth (α = 0.670) (Table 2).

#### Test–retest reproducibility

This evaluation was performed with 47 patients, and showed values ≥0.7 for the swallowing solids, swallowing liquids, dry mouth, mucus, teeth, speech, general pain and trismus domains and equal to 0.6 for the nutrition, mouth pain and taste/smell domains. The coefficients were low for neck and hearing items, 0.478 and 0.372, respectively (Table 6). Functionality, as measured by ECOG, remained stable across the two-time points (Kappa = 0.827; p < 0.001).

**Table 6:** Test retest reliability (5–16 days)

VHNSS 2.0 domains	ICC (N = 47)	95 % CI
Nutrition	0.600	(0.377–0.755)
Swallowing solids	0.809	(0.681–0.889)
Swallowing liquid	0.709	(0.530–0.827)
Dry mouth	0.797	(0.663–0.881)
Mouth pain	0.604	(0.388–0.758)
Mucus	0.854	(0.753–0.916)
Voice	0.802	(0.671–0.884)
General pain	0.751	(0.592–0.853)
Taste/smell	0.600	(0.384–0.755)
Teeth	0.718	(0.468–0.861)
Trismus	0.747	(0.590–0.851)
Neck	0.478	(0.224–0.671)
Hearing	0.372	(0.096–0.594)

*95* *% CI* 95 % confidence interval, *ICC* intraclass correlation coefficient

## Discussion

This study described stages in the validation process of the Brazilian Portuguese version of VHNSS 2.0, in a sample of patients with HNC being followed up. The results indicated that VHNSS 2.0 is an instrument with the potential to evaluate severity of oral changes associated with treatment, which includes radiotherapy of the head and neck region, for use in clinical practice and/or research in this population.

In general, the sociodemographic and clinical characteristics are representative of the study population because studies in the Brazilian population indicate that HNC prevalence is higher in men with low income, advanced-stage disease and squamous cell carcinoma histological type [24].

In this study, 161 (67.1 %) participants had less than 8 years of education, and all the subjects opted for the VHNSS 2.0 to be applied by the interviewer. However, the use of VHNSS 2.0 is feasible because the median time to respond, when applied by an interviewer, was 8 min (range 4–17 min), compared with 6 min (2–17 min) for EORTC H&N 35 and QLQ C30. In Brazil, it is known that there is a preference for assessment instruments to be applied by the interviewer [14]. Data from the validation process of the original version reported a time less than 10 min when self-administered [6]. In this study, the median time between the end of radiotherapy and the interview was 2 (range 0–23) years, whereas the median time of the original study was 1 (range 0–13) year [6]. Besides that, the VHNSS 2.0 has been tested before, during and up to 42 weeks post treatment [8]. Also noteworthy is that the number of items without responses was small and within the expected level.

When the frequency and severity of symptoms measured by VHNSS 2.0 were evaluated, the swallowing solids, dry mouth, mucus, taste/smell, voice and teeth domains had higher percentages of severe scores (score >7). For the dry mouth and difficulty chewing due to teeth/dentures items, 38.6 and 24.8 % of the population had severe levels (scores >7), respectively, compared with 36 and 16.4 % of patients, respectively, in a study published by Kolnick et al. [23]. The data of the validation process study of the original instrument showed significant percentages of moderate to severe scores in the swallowing solids, dry mouth, mucus, taste/smell, voice and general pain domains (>4) [7]. The presence of symptoms at considerable levels and severity reflects the need for monitoring, even in patients whose treatment is completed. The use of assessment instruments can provide useful information to help health professionals in patient care [5].

Regarding construct validity, the instrument was compared with the EORTC QLQ C30 and H&N 35, which, although evaluate QoL, have specific functional and symptom domains and are instruments with adequate psychometric properties for patients with HNC [25]. Thus, one might expect to find correlations higher than 0.4 between the items and assumed domains, and this has been confirmed, with the lowest correlation being 0.418 (VHNSS mouth pain × EORTC H&N 35 social eating) and the highest correlation being 0.756 (VHNSS 2.0 swallowing solids × H&N 35 swallowing), showing common features among these instruments.

It was not possible to discriminate among patient groups regarding any of the situations tested in four domains (swallowing liquid, mucus, hearing and neck). Differences were expected when comparing patients with different treatment completion times, since scores decrease over time but do not completely resolve [18, 26]. The analysis of the mean score of patients who had completed radiotherapy more than 12 months previously revealed that such scores were lower for this group, including in those domains where significant differences were not observed. However, contrary to these results, some studies, using questionnaires specifically developed for HNC (EORTC H&N 35), showed that at the 5-year follow up, a worsening of some symptoms, such as sense problems, less sexuality, dental problems, mouth opening and dry mouth, is revealed [27].

Using staging as a criterion for the discrimination among groups, the hypothesis was that patients with higher staging would have undergone more aggressive treatment, resulting in a greater symptom burden. A study of patients with HNC showed that patients whose disease was in stages III/IV had a higher symptom burden than those with stage I/II evaluated at 3 and 6 months, and this difference was less evident at 12 months [28]. When considering that the patients in our study are disease free, and that some have been followed up over many years, this difference was diluted in most domains, although the mean scores were higher in group III/IV than in group I/II.

Surgical criteria for group discrimination revealed significant differences in just three domains (swallowing solids, trismus and teeth). It was expected that patients undergoing combined therapeutic procedures would have higher mean scores, which was confirmed in most domains, although this was not statistically significant. According to Alicikus et al. [29], tumor site and therapeutic modality are the most important factors affecting QoL domains, including symptoms in treated HNC patients.

In an additional analysis comparing disease sites, the instrument could discriminate between patients whose disease was located in the oral cavity/oropharynx and hypopharynx/larynx in seven domains, with statistically significant differences.

Analyzing domains in terms of reliability, considering values ≥0.7 [16], the values in this study were satisfactory and ranged from 0.618 (nutrition) to 0.858 (swallowing solids). Those values were lower than those in the validation study of the original instrument, which ranged from 0.70 (swallowing liquid) to 0.95 (mucus) [7]. It is known that domains with small numbers of items and asymmetric distribution may have lower internal consistency [17].

The stability of the instrument, as measured by the ICC, proved to be satisfactory for most of the VHNSS 2.0 domains except for the hearing and neck items, where the values were much lower than expected, suggesting that there may be problems in these items that prevent proper understanding. Further studies are necessary to clarify the psychometric properties of the hearing and neck items in different populations and evaluate the need for instrument modifications.

The present study has some limitations. The selected patients were free of disease, which may have limited the retest to be performed in the recommended time interval for all patients, and may also have affected the known-groups analysis, in which the ability to discriminate between groups could not be validated for certain domains. This resulted in a large percentage of patients, in some items, with very low or absent symptomatology. Moreover, since all the subjects were follow up patients, this may hinder the generalization of the findings for patients undergoing treatment. So, further studies should be carried out in this group of patients.

Additionally, due to the choice of participants, the entire instrument was applied by the interviewer who read the items aloud, a procedure that may have facilitated the understanding of the instrument in a population with a mainly low education level. Hence, it would be important to confirm the psychometric properties, of the Brazilian Portuguese version, in self-administered situations. Another important limitation is that the assessment of responsiveness, to determine whether the instrument could detect changes over a time interval, was not done, and this would be important when the instrument is used in a clinical setting as an aid in the decision-making process. VHNSS 2.0 is an instrument available only in the English and Portuguese languages. Thus, there are few studies that have used this instrument to date, hindering any comparison of our findings with the literature.

## Conclusions

The validation process of the Brazilian Portuguese version VHNSS 2.0 has revealed that the instrument has adequate construct validity, as measured by convergent validity and known-groups analysis, and has acceptable internal consistency for most domains. Its use will contribute to the identification of symptoms and oral changes in patients with HNC who underwent exclusive or combined radiotherapy, thus allowing the development of strategies to monitor such changes. However, further studies are needed to assure that VHNSS 2.0 Brazilian Portuguese version is, beyond any reasonable doubt, a valid and reliable instrument to assess oral symptoms in HNC.

## Abbreviations

VHNSS 2.0: Vanderbilt Head and Neck Symptom Survey version 2.0
HNC: head and neck cancers
QoL: quality-of-life
NCI: National Cancer Institute
ICC: intraclass correlation coefficient
TNM: classification of malignant tumors
SSC: squamous cell carcinoma
ECOG: Eastern Cooperative Oncology Group
SD: standard deviation
R$: real
